# Active (Opt-In) Consent Underestimates Mean BMI-z and the Prevalence of Overweight and Obesity Compared to Passive (Opt-Out) Consent. Evidence from the Healthy Together Victoria and Childhood Obesity Study

**DOI:** 10.3390/ijerph15040747

**Published:** 2018-04-13

**Authors:** Claudia Strugnell, Liliana Orellana, Joshua Hayward, Lynne Millar, Boyd Swinburn, Steven Allender

**Affiliations:** 1 Global Obesity Centre, Centre for Population Health Research, Deakin University, 1 Gheringhap Street, Geelong, VIC 3220, Australia; josh.hayward@deakin.edu.au (J.H.); boyd.swinburn@auckland.ac.nz (B.S.); steven.allender@deakin.edu.au (S.A.); 2 Biostatistics Unit, Deakin University, Geelong, VIC 3220, Australia; l.orellana@deakin.edu.au; 3 Australian Health Policy Collaboration, Victoria University, Melbourne, VIC 8001, Australia; lynne.millar@vu.edu.au; 4 Australian Institute of Musculoskeletal Science, The University of Melbourne and Western Health, St Albans, VIC 3021, Australia; 5 School of Population Health, University of Auckland, Auckland 1142, New Zealand

**Keywords:** obesity prevalence, BMI, active consent, passive consent, non-participation bias, childhood, school

## Abstract

*Background:* Tracking population trends in childhood obesity and identifying target areas for prevention requires accurate prevalence data. This study quantified the magnitude of non-participation bias for mean Body Mass Index-z scores and overweight/obesity prevalence associated with low (opt-in) compared to high (opt-out) participation consent methodologies. *Methods:* Data arose from all Local Government Areas (LGAs) participating in the Healthy Together Victoria Childhood Obesity Study, Australia. Primary schools were randomly selected in 2013 and 2014 and all Grades 4 and 6 students (aged approx. 9–12 years) were invited to participate via opt-in consent (2013) and opt-out consent (2014). For the opt-in wave *N* = 38 schools (recruitment rate (RR) 24.3%) and *N* = 856 students participated (RR 36.3%). For the opt-out wave *N* = 47 schools (RR 32%) and *N* = 2557 students participated (RR 86.4%). Outcomes: differences between opt-in and opt-out sample estimates (bias) for mean BMI-z, prevalence of overweight/obesity and obesity (alone). Standardized bias (Std bias) estimates defined as bias/standard error are reported for BMI-z. *Results*: The results demonstrate strong evidence of non-participation bias for mean BMI-z overall (Std bias = −4.5, *p* < 0.0001) and for girls (Std bias = −5.4, *p* < 0.0001), but not for boys (Std bias = −1.1, *p* = 0.15). The opt-in strategy underestimated the overall population prevalence of overweight/obesity and obesity by −5.4 and −4.5 percentage points respectively (*p* < 0.001 for both). Significant underestimation was seen in girls, but not for boys. *Conclusions:* Opt-in consent underestimated prevalence of childhood obesity, particularly in girls. Prevalence, monitoring and community intervention studies on childhood obesity should move to opt-out consent processes for better scientific outcomes.

## 1. Introduction

Stemming the obesity epidemic is one of the greatest public health challenge for 21st century [1] with approximately 23% [2] of children and adolescents in developed countries overweight or obese. In high-income countries such as Australia, 20% of youths aged 5–17 years were classified as overweight and a further 7% as obese in 2014/15 [3]. These children and adolescents are at risk of the myriad of acute and chronic conditions associated with obesity [4], which typically persist into adulthood [5].

Difficulties in obtaining accurate and timely estimates of prevalence, trends and intervention effectiveness [6] present challenges to informed policy and service delivery. Several authors have made the case for routine, population-level surveillance/monitoring of anthropometric data among children [7], stressing the importance of sampling that accurately reflects the characteristics of the population under investigation [8]. Most studies among school-children require opt-in (active consent) from parents or guardians and achieve participation rates (PRs) between 30–60% [9]. Opt-out (passive consent) recruitment methods achieve higher PRs [9]; for example the English National Child Measurement Program (NCMP) mandated all children in government schools in reception (aged 4–5 years) and year 6 (aged 10–11 years) have their height and weight measured using an opt-out consent procedure [10]. Beginning in 2006/07, the NCMP currently measures over 1 million primary school children annually with PRs above 93% [10].

This heterogeneity in PRs raises questions about non-participation bias; a bias that occurs when those who consent to participate are a sub-population which is not representative of the broader study population. Examination of the magnitude of non-participation bias is hampered by the limited ability to obtain information on those who declined participation. Previous attempts to assess non-participation bias in school-based obesity studies include comparing respondents and non-respondents’ demographic characteristics (e.g., age, gender, socioeconomic position) and collecting teachers’ estimates of non-participants’ heights and weights [11]. The two studies of non-participation bias in school-based childhood obesity research report contrasting findings. Analyses of several waves of the census-styled (all primary schools involved) England’s NCMP found obesity among Grade 6 students to be underestimated by 1.3% for 2006/07 (PR = 78%), 0.8% for 2007/08 (PR = 87%) and 0.7% for 2008/09 (PR = 89%) [10]. In contrast, an opportunistic analysis of the Healthy Kids project within five schools (elementary, primary and secondary) in Anardarko, Oklahoma found no significant difference in weight status of opt-in consent (769/2053; PR = 37.0%) in 2001/02 compared to an opt-out consent model in 2002/03 (1895/1960; PR = 96.7%) [12]. Although the study found younger children (Prep-Grade 5) compared to older children (Grade 9–12) and female students had significantly higher odds of participation under an opt-in consent model [12]; highlighting possible age and gender-specific influences on participation rates.

Accurate assessment of non-participation bias is difficult because seldom are the same sampling methods repeated within short time frames with the only variation being in recruitment method. Here we report one such example where the only methodological change between two subsequent waves of the study was a shift from opt-in consent in 2013 to opt-out consent in 2014. Herein we examined the magnitude of non-participation bias on estimates of overweight and obesity prevalence and mean Body Mass Index z-score (BMI-z). We hypothesized that, compared to opt-out consent, opt-in consent would produce lower participation rates and result in underestimates of overweight and obesity prevalence and mean BMI-z.

## 2. Methods

### 2.1. Setting and Study Design

This repeat cross-sectional study examined changes in BMI-z and associated risk factors of primary school children in Grade 4 and Grade 6. Schools were selected from 26 Local Government Areas (LGA) that formed the embedded cluster randomized control trial within the Healthy Together Victoria initiative. Detailed descriptions of the study design, sampling approach, sample size, evaluation measures and protocol are reported elsewhere [13]. Herein, a brief overview of the methodology utilized for the current manuscript is provided.

### 2.2. School Sampling and Participant Recruitment

Of the 79 LGAs in Victoria, Australia, 26 LGAs were randomly selected as part of a cluster randomized controlled trial. Within each LGA, three schools were selected at random and invited to participate from a list of all primary schools. Where all three schools declined participation, the next three primary schools were invited, until at-least one school per LGA consented. Principals were sent written invitations to participate with follow-up phone calls, emails and/or visits to the school. In consenting schools, all students in Grades 4 (approx. 9–10 years old) and 6 (approx. 11–12 years old) were invited to participate via a plain language statement and consent form. In 2013 an opt-in recruitment procedure was used, with students who returned a consent form signed by their parent/guardian enrolled in the study. In 2014, an opt-out consent procedure was used whereby students were enrolled unless they returned a signed opt-out form from parents/guardians or the student did not agree to participate either previously or on the day of data collection. Other than the change from opt-in to opt-out consent, an identical methodology was used in 2013 and 2014 to recruit participants and collect data. In 2013, data collection occurred during Term 4 (Monday 7 October–Friday 20 December 2013) and in 2014, during Term 3 (Monday 14 July–Friday 19 September 2014). The change in school term was due to feedback from school principals that Term 4 was overburdened with other commitments.

### 2.3. Measures

Participants had their height, weight and waist measured by trained researchers and completed a behavioural questionnaire on physical activity, sedentary behaviour, diet quality and wellbeing [13]. There were no exclusion criteria for participation. Measurements were taken throughout the school day by trained research staff in a professional, private and respectful manner.

*Demographic characteristics:* Date of birth, gender, residential postcode, language spoken at home, country of birth and ancestry were collected through a self-reported questionnaire. For this study, school postcode was used to categorise individuals within quintiles of Relative Socio-Economic Advantage and Disadvantage which was derived from the Australian Bureau of Statistics (ABS) Socio-Economic Indexes for Areas (SEIFA) index, 2011 Australian Census [14]. In addition, the schools’ Index of Community Socio-Educational Advantage (ICSEA) developed by the Australian Curriculum, Assessment and Reporting Authority was collected (https://www.myschool.edu.au) [15]. The ICSEA represents the level of educational advantage at the school-level and aims to enable fair and meaningful comparisons of literacy and numeracy performance of students within a school with that of schools serving students with similar backgrounds [15].

*Anthropometry:* Height was measured to the nearest 0.1 cm using a portable stadiometer (Charder HM-200P Portstad, Charder Electronic Co Ltd., Taichung City, Taiwan) and weight to the nearest 0.1 kg using an electronic weight scale (A&D Precision Scale UC-321; A7D Medical, San Jose, CA, USA) without shoes and whilst wearing light clothing. Age and sex-specific body mass index (BMI) z-scores were calculated using the World Health Organizations’ (WHO) growth reference [16]. Definitions of overweight or obesity (≥+1 standard deviation) and obesity (≥+2 standard deviation) were calculated according to the BMI z-score for age using the WHO growth reference [16]. The average of two measurements of height and weight was calculated unless a third measure was needed when a discrepancy of 0.5 cm or 0.5 kg occurred, the average of all three measurements was used in these instances.

The study was approved by Deakin University’s Human Research Ethics committee (2013-095), the Victoria Department of Education and Training (2013_002013), and the Catholic Education Offices of Melbourne, Sale, Sandhurst and Ballarat (Healthy Together Children’s Evaluation).

### 2.4. Data Analysis

The target populations for this study consisted of children attending Grade 4 and Grade 6 in schools located in a selected set of LGAs of Victoria, Australia, in 2013 and 2014. Analyses were based on two assumptions: (1) that the distribution of the outcomes of interest (BMI-z and weight status) were identical in the 2013- and 2014-population; and (2) that the sample of children/families who were invited to participate in 2013 can be thought as similar/equivalent to the sample of children/families invited to participate in 2014. The first assumption is highly plausible because population-level BMI-z is unlikely to have changed significantly over the 9-months between study waves [17]. The second assumption is supported by the fact that in both years, schools were randomly selected from the school list in the 26 participant LGAs and the enrolment and recruitment procedures for schools and students were identical. The assumption was further assessed comparing schools characteristics between waves: school-level ICSEA; school-level SEIFA; number of children enrolled in Grade 4, Grade 6 and all primary levels (Wilcoxon’s test); type of school (government, independent or Catholic); and location of the school as located in major cities, inner regional or outer regional (Chi-squared or Fisher’s exact test). Significance was set at *p* < 0.05.

Under the former assumptions the children invited to participate under the opt-out and opt-in strategies are random samples from the same underlying population. Each sample provides an estimate of the mean (or prevalence) of the outcome of interest in this population, for example, BMI score (denoted as ${\bar{X}}_{opt-in}$ and ${\bar{X}}_{opt-out}$). Therefore any difference between estimates from the two samples that can’t be attributed to random chance is evidence of non-participation bias due to the different consent strategies.

We denote $n_{opt-in}$ and $n_{opt-out}$ the number of children under each consent strategy ($n_{opt-in}<n_{opt-out}$). We used a resampling strategy to explore the estimates obtained in samples of size $n_{opt-in}$ selected from the set of children in the opt-out wave. A total of 10,000 simple random samples were selected without replacement from the opt-out set of children and the statistics of interest, for example, mean BMI-z score, was calculated in each one of the 10,000 samples. These values were used to: (1) produce the sampling distribution of the statistics (histogram); (2) estimate the variability of the statistics, that is, the standard error (SE); and (3) calculate the percentage of random samples with a value of the statistics equal to or more extreme than the value ${\bar{X}}_{opt-in}$ observed in the opt-in sample (P_resampling_). A small P_resampling_ value provides evidence of non-participation bias, indicating that the sample obtained under opt-in consent is not “representative” of the larger sample obtained under opt-out consent. For simplicity, we will refer to P_resampling_ as P.

Bias estimates are presented for mean BMI-z-score, prevalence of obesity/overweight and prevalence of obesity. The estimates were calculated for the whole data set and for the subpopulations defined by gender, gender and Grade (4 and 6), and gender and location (major cities, inner regional, outer regional).

## 3. Results

School participation rates were similar in both study waves (24.4% vs. 32.0%; NS), while student participation rate was 36.3% under opt-in and 84.4% under opt-out consent (*p* < 0.001) (Table 1). Characteristics of participating schools did not differ between the two study periods (Table 2).

**Figure 1:**
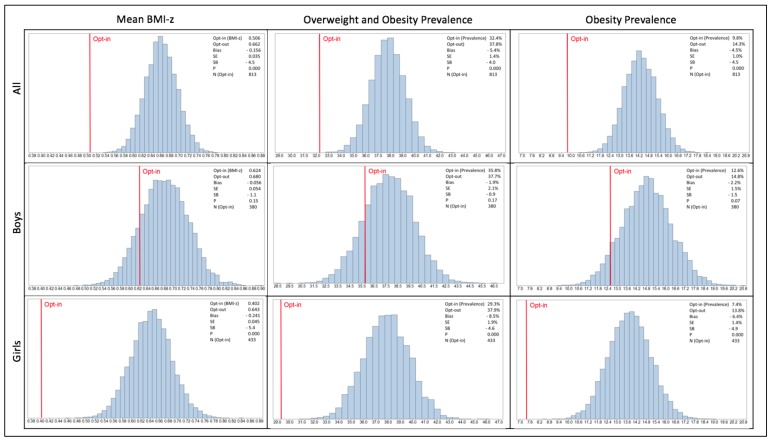
Sampling distribution of the mean Body Mass Index z-score (BMI-z) score, prevalence of overweight and obesity and prevalence of obesity estimates based on 10,000 samples selected from the opt-out data, overall and by gender. Histograms represent the sampling distribution of each estimate over 10,000 random samples drawn from the opt-out sample. N(opt-in) = selected random sample size; SE = standard error estimated under resampling; SB = standardized bias = bias/SE; Red line indicates the estimate based on the data collected under opt-in consent; P = percentage of random samples with a value of the statistics equal to or more extreme than the value opt-in estimate.

The sampling distribution of mean BMI-z, prevalence of overweight/obesity and prevalence of obesity overall and for boys and girls based on 10,000 random samples of size $n_{opt-in}$ from the opt-out data sets is shown in Figure 1. The opt-in estimate (red line) was consistently lower than the opt-out estimate (mid-point of the sampling distribution). For the whole sample, the mean BMI-z score, estimated under opt-in strategy was “outside” the range of means obtained when resampling from the opt-out sample, that is, lower than the mean calculated for any of the 10,000 samples. The same conclusion is true for overweight/obesity and obesity prevalence in the whole sample. Non-participation bias was particularly noticeable within girls.

There was strong evidence of non-participation bias for BMI-z overall (Std bias = −4.5, *p* < 0.0001) and for girls overall (Std bias = −5.4, *p* < 0.001) and girls in Grade 4 (Std bias = −5.9, *p* < 0.0001) (Table 3). Opt-in consent underestimated overweight/obesity prevalence in girls by 8.5 percentage points (*p* < 0.0001) and obesity prevalence by 6.4 percentage points (*p* < 0.0001). Negative bias attributable to opt-in consent was evident by region and gender with the exception of boys in major cities. In boys in major cities, there was no evidence of bias due to opt-in consent.

## 4. Discussion

Compared to opt-out consent, opt-in consent methods resulted in significant underestimation of overweight/obesity (−5.4 percentage points) and obesity (−4.5 percentage points) for boys and girls. Additionally, the magnitude of underestimation was significant for girls but not boys; although patterns of underestimation were evident for inner and outer regional boys and younger girls. These findings indicate that lower participation rates associated with opt-in consent could mask the prevalence of combined overweight/obesity and particularly obesity by as much as 8.5 percentage points.

These results demonstrate that participants who opt-in to school-based childhood obesity measurement programs are significantly different (more likely of healthier weight) than those recruited using opt-out consent. The magnitude of this non-participation bias was greater among girls compared to boys, which may reflect differential social pressures around body image, girls’ greater pre-occupation with weight, concerns about dieting, and drive for thinness compared to boys’ during childhood [18]. Studies in other countries have observed that parental concern regarding child weight was a significant barriers to consent in obesity surveillance studies [19]. These observations appear to interact with age; in our study we observed higher non-participation bias among younger (Grade 4) compared to older girls (Grade 6). Mellor, Rapoport & Maliniak (2008) support these observations reporting that parents of younger (Grade 3) children and parents of children with overweight or obesity were significantly more likely to opt-out their child compared to older (Grade 6 and 7) and healthier weight peers, though no significant gender differences were observed [20].

Caution should be taken in assuming that improving overall response rates will automatically improve sample representativeness. In our study, the largest bias was not necessarily related to the lowest participation rates. As an example, obesity prevalence bias was larger among boys from inner regional and remote areas, as compared to city-dwelling peers, even though participation rates were almost double among the first subgroup (opt-in participation relative to opt-out: outer regional = 54% vs. city-dwelling = 29%). Improving the overall response rate would not necessarily improve the sample representativeness with regards to weight status as a study with a reasonably high response rate can still be subject to participation bias if non-participants are systematically different from those who participated in the study.

This study extends lessons from the NCMP in England [10] to provide the first examination of non-participation bias for an Australian sample and to consider the contribution of remoteness. Our results generally supports findings of the NCMP and contrasts with the much smaller Anardarko (Study) (3 primary, 1 elementary, 1 high school) set in a single rural city which found no participation bias [12].

### 4.1. Strengths and Weaknesses of the Study

The maintenance of identical protocol, with the exception of a change from opt-in to opt-out consent, in subsequent studies 9 months apart provide a unique data set to examine the influence of non-participation bias on estimates of BMI-z and overweight and obesity prevalence. Comparability of schools was further supported by their similar demographic characteristics supporting the contention that observed differences were not attributable to school-level differences between study waves.

Our resampling approach underestimates the standard error of the bias in two ways. First, it does not account for the variability in the opt-out estimates, the opt-out sample played the role of a “fixed” population in this approach. The rationale was that the opt-out sample, with >80% participation rate, is a large and well representative sample of the children population that we can expect to reach in a school-based survey in Victoria, Australia. Second, when resampling, we selected samples without accounting for the clustering induced by schools because the schools enrolled in the two waves were different and sampling inside schools was not possible. Of note, the intraclass correlation coefficient was generally small (e.g., BMI-z overall and boys 0.020, girls 0.028). We chose the resampling approach instead of a standard two sample comparison under a multilevel model because it graphically highlights the “non-representativeness” of the opt-in sample especially for some sub-populations. One of the limitations of our study is that our estimates might be influenced by the fact that data collection occurred in different school terms for 2013 and 2014. In England, month of measurement was associated with BMI-z among very young children (aged approx. 5–6 years) and a decline in BMI z-score of −0.00788 every month compared to September (Spring) as the reference month [21]. However, neither in England nor Australia has this month effect been reported in older children. Finally, the magnitude of the bias reported here for some subgroups is extremely large and it is this which is most relevant for population level studies.

### 4.2. Implications for Policy and Practice

This large sample of primary school children has demonstrated significant non-participation bias between opt-in and opt-out consent methods. The differences in estimates should not be assumed as standard for comparing studies employing these consent methods in other populations but our study does suggest that opt-out consent methods will provide higher participation rates and more reliable estimates than more traditional methods. These results would indicate that estimates of population prevalence and trends over time require large scale data with high participation which in Australia can only be realistically achieved with opt-out consent.

These results indicate that comparison of samples with differing participation rates under an opt-in model will have inherent bias on estimates of BMI-z and overweight/obesity due to low participation rates and should be interpreted with caution. The study also suggests that studies conducted under alternate opt-in and opt-out consent procedures over time will have fundamentally different samples, limiting the comparability of the estimates.

These findings also have implications for understanding intervention effectiveness. That opt-in participants are more likely to be healthier than a general population sample leads to the caution that any observed effects are drawn from an already healthier sample. Therefore trial outcomes based on opt-in consent cannot be generalized to the whole population as they may overestimate or underestimate the intervention effects depending on whether the intervention had a greater or lesser impact on those children who were already overweight or obese and would not participate in measurements with opt-in consent processes.

The findings also strengthen the imperative for high quality anthropometric surveillance under an opt-out model. While contentious in some countries, surveillance programs are used in Arkansas, USA [22], and by the NCMP in England, with no adverse effects on participants in local community-based obesity prevention trials observed [23,24].

Based on this evidence, assessments of school-based anthropometry studies by ethics committees and Internal Review Boards should factor in the higher chances of better science outcomes without adverse effects using the opt-out consent process. 

## 5. Conclusions

The standard practice of opt-in consent produces biased population estimates of childhood overweight and obesity prevalence. Opt-out consent is essential to provide accurate estimates of current obesity prevalence and trends and to understand effectiveness of community childhood obesity prevention interventions.

## Figures and Tables

**Table 1:** Student participant characteristics by consent methods.

	**Opt-In**	**Opt-Out**	
Invited Schools, *n*	156	147	
Participating schools, *n* (response rate)	38 (24.4%)	47 (32.0%)	*p* = 0.14 ^a^
Eligible students (Grade 4 & 6), *n*	2357	2959	
Participating children, *n* (response rate)	856 (36.3%)	2557 (84.4%)	*p* < 0.0001 ^a^
Participating children per school			
Mean (SD)	22.5 (15.5)	54.4 (48.1)	*p* = 0.0005 ^b^
Minimum—Maximum	3—65	2—212	
Enrolled children with anthropometric data	**Opt-In** ***n* (%)**	**Opt-Out** ***n* (%)**	**Opt-In/Opt-Out**
Total	813	2419	34%
Boys	380 (47)	1228 (51)	31%
Girls	433 (53)	1191 (49)	36%
Boys Grade 4	194 (51)	636 (52)	31%
Grade 6	186 (49)	592 (48)	31%
Girls Grade 4	233 (54)	611 (51)	38%
Grade 6	200 (46)	580 (49)	34%
Boys Major cities	191 (50)	667 (54)	29%
Inner regional	127 (33)	445 (36)	29%
Outer regional	62 (16)	116 (9)	53%
Girls Major cities	204 (47)	620 (52)	33%
Inner regional	173 (40)	468 (39)	37%
Outer regional	56 (13)	103 (9)	54%

^a^ Chi-squared test; ^b^ Kruskal-Wallis test.

**Table 2:** Comparison of characteristics of participating schools.

	Opt-In (*n* = 38)	Opt-Out (*n* = 47)	
	Mean (SD)		*p*-value ^¥^
Index of Community Socio-Educational Advantage (ICSEA)	989 (46)	980 (60)	0.48
Socio-Economic Index for Areas (SEIFA)	968 (54)	960 (54)	0.51
Enrolment Grade 4	37 (34)	39 (37)	0.80
Enrolment Grade 6	34 (28)	38 (35)	0.60
Enrolment (total)	272 (238)	342 (385)	0.31
	No. (%)		*p*-value ^#^
Government school	31 (82)	38 (81)	0.98
Location			1.00
Major cities	14 (37)	18 (38)	
Inner Regional	16 (42)	20 (43)	
Outer Regional	8 (21)	9 (19)	

^¥^ Wilcoxon test; ^#^ Fisher’s exact test.

**Table 3:** Resampling estimates of opt-in bias based on 10,000 samples selected from the opt-out data, by gender, gender/grade-level and gender/location index.

		BMI-z Score Mean						Overweight and Obesity Prevalence						Obesity Prevalence					
		Opt-In	Opt-Out	Bias	SE	Std Bias	*p*	Opt-In	Opt-Out	Bias	SE	Std Bias	*p*	Opt-In	Opt-Out	Bias	SE	Std Bias	*p*
All		0.506	0.662	−0.156	0.035	−4.5	<0.0001	32.4	37.8	−5.4	1.36	−4.0	<0.0001	9.8	14.3	−4.5	1.00	−4.5	<0.0001
Boys		0.624	0.680	−0.056	0.054	−1.1	0.15	35.8	37.7	−1.9	2.07	−0.9	0.17	12.6	14.8	−2.2	1.53	−1.4	0.070
Girls		0.402	0.643	−0.241	0.045	−5.4	<0.0001	29.3	37.9	−8.5	1.86	−4.6	<0.0001	7.4	13.8	−6.4	1.31	−4.9	<0.0001
Boys	Grade 4th	0.704	0.728	−0.024	0.077	−0.3	0.38	39.2	38.1	1.1	2.9	0.4	0.35	14.4	15.6	−1.1	2.19	−0.5	0.31
	Grade 6th	0.541	0.628	−0.088	0.074	−1.2	0.12	32.3	37.3	−5.1	2.96	−1.7	0.050	10.8	14	−3.3	2.10	−1.6	0.054
Girls	Grade 4th	0.379	0.734	−0.355	0.061	−5.9	<0.0001	27.9	40.8	−12.9	2.56	−5.0	<0.0001	7.7	16	−8.3	1.86	−4.5	<0.0001
	Grade 6th	0.428	0.547	−0.119	0.066	−1.8	0.039	31.0	34.8	−3.8	2.65	−1.4	0.077	7	11.4	−4.4	1.84	−2.4	0.0076
Boys	Major cities	0.720	0.609	0.111	0.082	1.4	0.086	42.4	35.8	6.6	3.07	2.1	0.017	14.1	13.8	0.3	2.18	0.1	0.43
	Inner regional	0.547	0.745	−0.197	0.084	−2.4	0.0088	29.1	39.6	−10.4	3.56	−2.9	0.0015	11.8	15.1	−3.3	2.62	−1.3	0.098
	Outer regional	0.487	0.841	−0.354	0.102	−3.5	0.0001	29	41.4	−12.4	3.84	−3.2	0.0012	9.7	19.8	−10.1	3.25	−3.1	0.0012
Girls	Major cities	0.369	0.580	−0.212	0.066	−3.2	0.0003	29.4	38.1	−8.7	2.8	−3.1	0.0007	5.9	11.5	−5.6	1.82	−3.1	0.0007
	Inner regional	0.482	0.743	−0.261	0.071	−3.7	<0.0001	31.2	39.1	−7.9	2.9	−2.7	0.003	10.4	17.1	−6.7	2.28	−2.9	0.0012
	Outer regional	0.276	0.567	−0.291	0.094	−3.1	0.0004	23.2	31.1	−7.9	4.43	−1.8	0.029	3.6	12.6	−9.1	3.13	−2.9	0.0008

SE = standard error estimated under resampling; Std bias = standardized bias = bias/SE; *p* = percentage of random samples with a value of the statistics equal to or more extreme than the opt-in estimate.
